# Epidemiology of *Kudoa septempunctata* food poisoning in Japan from 2013 to 2023

**DOI:** 10.1038/s41598-026-38632-2

**Published:** 2026-02-09

**Authors:** Yoshiro Hadano, Hirotake Mori, Yuichiro Tanaka, Aongart Mahittikorn, Satoshi Ohno

**Affiliations:** 1 Division of Infection Control and Prevention, Shimane University Hospital, 89-1 Enyacho, Izumo, Shimane 693-8501 Japan; 2 Department of General Medicine, Faculty of Medicine, Juntendo University, Tokyo, Japan; 3 Faculty of Medicine, Shimane University, Izumo, Shimane Japan; 4 Department of Protozoology, Faculty of Tropical Medicine, Mahidol University, Bangkok, Thailand; 5 Clinical Research Center, Shimane University Hospital, Izumo, Shimane Japan

**Keywords:** *Kudoa septempunctata*, Japan, Epidemiology, Food poisoning, Infectious diseases, Epidemiology

## Abstract

*Kudoa septempunctata*, a parasite found in olive flounder, poses a growing food safety risk in East Asia, particularly in Japan and South Korea. *K. septempunctata* poisoning caused by raw fish consumption causes brief gastrointestinal symptoms. However, long-term, national-scale aggregated epidemiological data for *K. septempunctata* food poisoning are limited. In this retrospective study, we examined the recent epidemiological trends and characteristics of *K. septempunctata* food poisoning cases reported in Japan between January 2013 and December 2023. Ministry of Health “Foodborne Illness Statistical Data” were assessed for case counts, outbreaks, and implicated foods. Reported cases totaled 2009, reaching a peak in 2014 (429 cases) then declining to < 100 cases during the COVID-19 pandemic. October had the highest number of monthly reports. Flounder, particularly sashimi and sushi, were implicated in 99% of cases. The highest case counts occurred in Yamaguchi, Osaka, and Fukuoka prefectures (160, 155, and 154, respectively). Tottori, Shimane, Yamaguchi and Oita prefectures had the highest incidence rates (14.3, 10.9, 10.7, and 10.7 per 1,000,000 population, respectively). Prefectures along the Sea of Japan typically reported higher incidence rates. This study highlights the importance of continued surveillance and reporting of *K. septempunctata* poisoning, and the need to consider *Kudoa* infections in the differential diagnosis of food poisoning cases involving raw fish consumption.

**Supplementary Information:**

The online version contains supplementary material available at 10.1038/s41598-026-38632-2.

## Introduction

*Kudoa septempunctata*, a myxosporean parasite, has emerged as a significant concern for food safety and public health, particularly in East Asian countries such as Japan and South Korea^1–4^. This parasite, which infects the muscle tissue of olive flounder (*Paralichthys olivaceus*), has been identified as a novel causative agent of foodborne illnesses associated with the consumption of raw fish^5^. The infection typically manifests as gastrointestinal symptoms, including diarrhea and vomiting, that develop within 12 h of ingestion of contaminated food then generally subside within a few days^2,3^. *K. septempunctata* is the second most common cause of parasitic food poisoning in Japan after *Anisakis* species^6^.

To date, approximately 100 *Kudoa* species have been identified, although the life cycles and transmission routes have been clarified for only a small subset^7,8^. Since 2003, food poisoning cases associated with raw fish, particularly raw *P. olivaceus*, have increased in Japan, averaging over 100 cases annually and peaking at 158 in 2010^2^. Despite extensive testing, no bacteria, viruses, toxins, or chemicals were detected in the implicated fish; however, the pathogen was subsequently confirmed as *K. septempunctata* through epidemiological, metagenomic, and animal analyses^2,8^. Recent years have seen a growing number of cases involving vomiting and diarrhea of unknown origin occurring within a few hours of consuming raw fish^2^. According to the Japanese Ministry of Health, Labour and Welfare (MHLW), 135 of 198 food poisoning cases of unknown origin recorded between June 2009 and March 2011 were linked to the consumption of olive flounder^9^. Subsequent investigations in June 2011 identified *K. septempunctata* as a novel cause of food poisoning^10^. Considering the increasing incidence of *K. septempunctata* food poisoning cases^9,11,12^ and the commercial importance and widespread distribution of olive flounder in Japan, these outbreaks represent an increasing public health concern.

Significant gaps remain in our understanding of the epidemiological characteristics of food poisoning attributed to *Kudoa* species. Despite previous clinical studies on outbreaks caused by this species in Japan and South Korea^2–4^, detailed national-level aggregated occurrence data remain limited. Therefore, the aim of this study is to characterize the recent epidemiological trends and characteristics of *K. septempunctata* food poisoning using national-level data from Japan.

## Materials and methods

This retrospective study was conducted from January 2013 to December 2023. The study protocol was approved by the institutional review board of Shimane University Hospital (no. 20250116-1). Owing to the retrospective nature of the study, the institutional review board waived the need to obtain informed consent.

Under the MHLW Food Sanitation Act, which classifies *Kudoa* food poisoning as a form of parasitic food poisoning, medical doctors in Japan are legally required to report suspected cases of food poisoning to local public health centers. Individuals with food poisoning are defined as “individuals who have been poisoned, or those suspected of being poisoned, because of exposure to contaminated food, additives, utensils, or containers and packaging”^6^. Public health centers then investigate reported cases to identify the causative food, etiological agent, or facility. If a public health center receives an individual complaint or report of foodborne illness after eating at a restaurant, it is investigated and treated as a food poisoning case^6^. Diagnostic tests for *K. septempunctata* are challenging to conduct in most hospitals and typically performed at local public health centers or research institutes. Generally, the causative agents of food poisoning are confirmed by detecting pathogens in leftover food or food samples consumed by the patient, as well as by polymerase chain reaction (PCR) testing of the patient’s stool^7,13^. Other approaches include microscopic examination methods for counting spores from flounder samples, genetic testing methods such as PCR and loop-mediated isothermal amplification, and immunoassay kits^13^.

In this study, cases of *K. septempunctata* food poisoning were extracted from the “Foodborne Illness Statistical Data” report for the period spanning January 1 2013 to December 31 2023, obtained from the MHLW website^6^. We analyzed annual and monthly numbers of reported cases, food poisoning incidents, implicated foods, involved facilities, numbers of contacts (persons who consumed the implicated food, regardless of symptom development), and affected patients at the national and prefectural levels. Incidence rates were calculated as the average annual number of cases per 1,000,000 population. Population data for each year were obtained from the “e-Stat” report on the MHLW website^14^. All data used in this study are publicly available from official government sources.

To create a map of Japan for visualizing the distribution of *K. septempunctata* food poisoning cases, we used R programming language and associated packages. Specifically, the “rnaturalearth” package was employed to obtain geographical data for Japan’s prefectures^15^, and “ggplot2” was used to create heat maps^16^.

To assess the potential impact of the coronavirus disease 2019 (COVID-19) pandemic on reported cases, the study period was divided into three phases: phase 1 (2013–2019), before the COVID-19 pandemic; phase 2 (2020–2022), during the COVID-19 pandemic and the implementation of preventive measures; and phase 3 (2023), following the loosening of preventive measures. The COVID-19 pandemic began at the end of December 2019 and spread worldwide, requiring hospitals in Japan and other countries to respond to this situation^17,18^. As of May 2023, the Japanese government no longer requested individuals to adhere to standardized basic infection control measures in daily life, and individuals with COVID-19 or close contacts of patients with COVID-19 were no longer requested to refrain from going out^19^.

### Statistical analyses

A Poisson regression model was applied to assess the association between trends of *K. septempunctata* food poisoning cases per year and study phase. To adjust for population size, the logarithm of the population size was included as an offset variable along with time. Two-tailed *p*-values of < 0.05 were considered statistically significant. Statistical analyses were performed using R software (version 4.3.0; R Foundation for Statistical Computing, Vienna, Austria).

## Results

Between January 2013 and December 2023, 2,009 cases of *K. septempunctata* poisoning were reported (Table 1). The majority of cases occurred among older adults, with individuals aged 60–69 years (23.5%) and 70 years and older (26.0%) together accounting for almost half of all cases. Cases among individuals younger than 20 years comprised less than 2.5% of all cases. Females accounted for 55.9% of cases, with a notable dominance observed in older age groups.

**Table 1:** Distribution of reported cases by age group and sex.

Age group	Total	Male	Female
0–4	1	0	1
5–9	3	2	1
10–14	10	6	4
15–19	11	6	5
20–29	119	46	73
30–39	180	100	80
40–49	290	144	146
50–59	392	179	213
60–69	472	192	280
70+	522	207	315
Unknown	9	4	5
Total	2009	886	1123

**Fig. 1:**
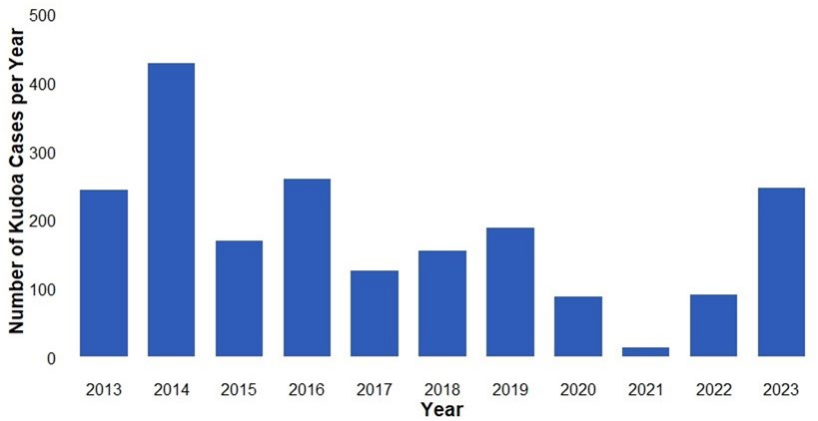
Total number of *Kudoa septempunctata* food poisoning cases in Japan from 2013 to 2023.

**Fig. 2:**
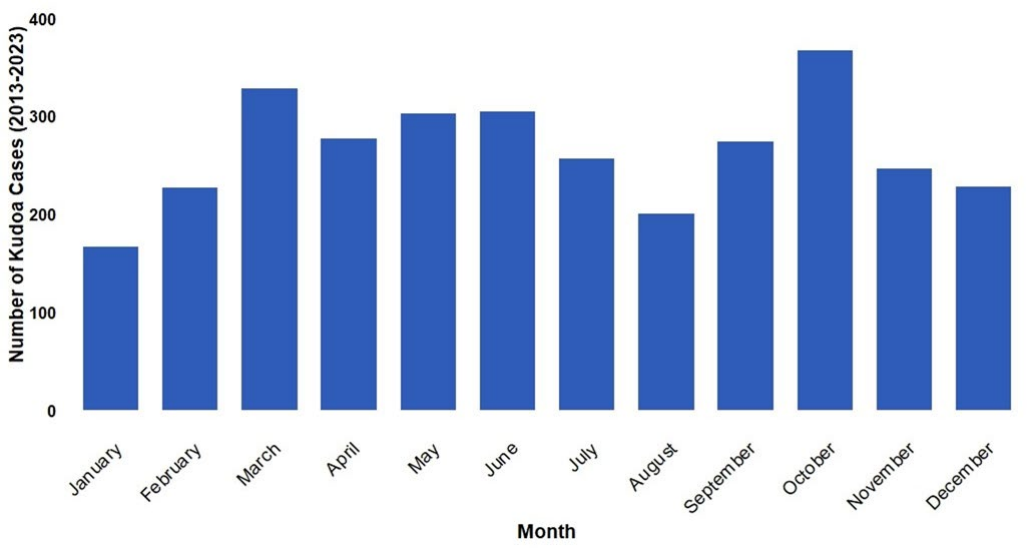
Seasonal diagnosed cases of *K. septempunctata* food poisoning in Japan from 2013 to 2023.

The annual number of cases peaked in 2014 (429 cases and 43 outbreaks of food poisoning), ranged between 100 and 200 cases per year thereafter, and declined to fewer than 100 cases in 2020 (Fig. 1). The number of cases had been decreasing by 12% annually before the COVID-19 pandemic (phase 1); however, during the pandemic (phase 2), the number of cases declined significantly (incidence rate ratio: 0.54 [95% confidence interval: 0.39–0.74]; Table 2). In 2023 (phase 3), the number of cases began to increase again. The number of food poisoning outbreaks followed a similar pattern (Supplementary Fig. S1). The average number of *K. septempunctata* food poisoning outbreaks was 17.5 per year (minimum: four in 2021; maximum: 43 in 2014). The number of monthly case reports was highest in October, followed by March, although cases were reported throughout the year (Fig. 2). Among the implicated foods, 99% included flounder, particularly sashimi and sushi.

**Table 2:** Parameters of the Poisson regression model describing the relationship between *K. septempunctata* food poisoning incidence and the COVID-19 pandemic.

Variables	Coefficient	Incidence rate ratio (95% CI)	*p*-value
Time (in years)	− 0.12	0.88 (0.87, 0.91)	< 0.01
During the COVID-19 pandemic	− 0.62	0.54 (0.39, 0.74)	< 0.01
After the COVID-19 pandemic	0.97	2.64 (2.34, 2.97)	< 0.01

CI: confidence interval. COVID-19: coronavirus disease 2019.

**Fig. 3:**
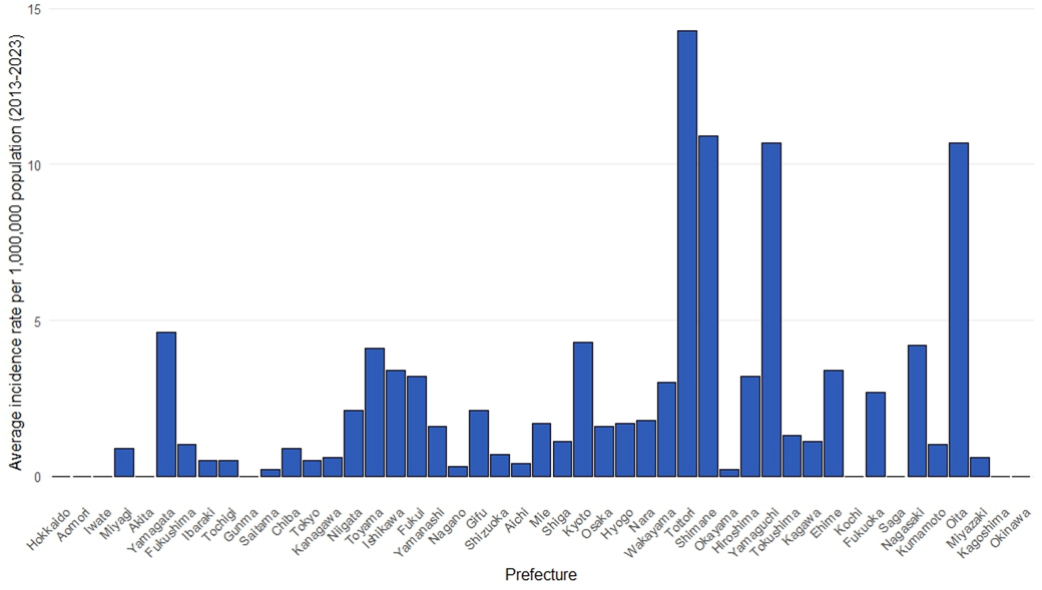
Average number of *K. septempunctata* food poisoning cases per 1,000,000 population reported in Japan each year by prefecture (2013–2023).

**Fig. 4:**
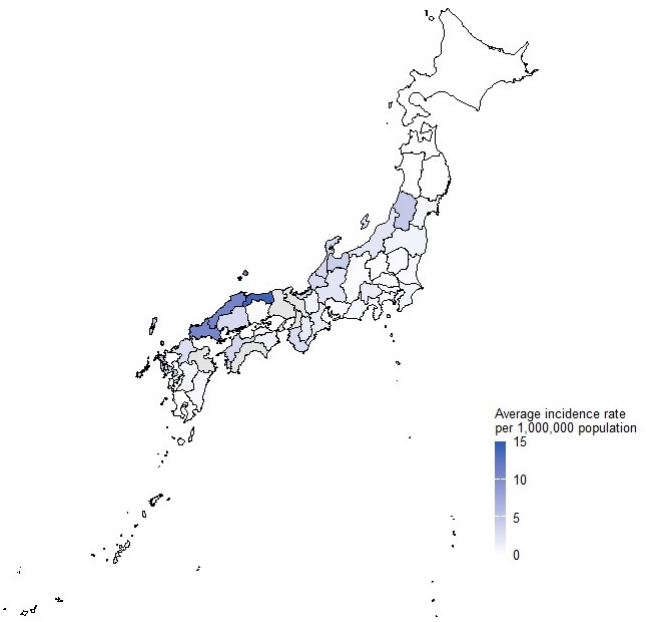
Geographic heat maps of the average number of *K. septempunctata* food poisoning cases reported each year in Japan (2013–2023).

The prefectures with the highest total number of cases from 2013 to 2023 were Yamaguchi, Osaka, and Fukuoka (160, 155, and 154, respectively) (Supplementary Fig. S2). For the same period, the prefectures with the highest average incidence rates per 1,000,000 population were Tottori, Shimane, Yamaguchi, and Oita (14.3, 10.9, 10.7, and 10.7, respectively) (Fig. 3). Western Japan and coastal areas along the Sea of Japan tended to have higher average incidence rates (Fig. 4).

## Discussion

Following recent reports of *K. septempunctata* food poisoning in Japan, this study provides a long-term nationwide overview of the epidemiology of *K. septempunctata* food poisoning in Japan from 2013 to 2023.

Overall, the number of cases and outbreaks declined slightly over time, peaking in 2014. After the *K. septempunctata* food poisoning outbreak in 2011, measures were implemented for farmed flounder, including ultraviolet treatment of rearing water during the seed production process, and thorough PCR and microscopic inspections during both introduction and shipment stages at aquaculture farms. For imported flounder, the government also introduced quantitative PCR testing at custom checkpoints^12,20^. Our results suggest that these measures were effective. During the COVID-19 pandemic, the number of *K. septempunctata* food poisoning cases tended to decrease. Similar reductions in enteric infections were reported in several countries, including Japan^21–23^, likely due to reduced dining out, improved hygiene practices in food handling, travel restrictions, and shifts in food consumption patterns, including increased consumption of home-cooked meals and fewer opportunities to eat out or consume raw fish. These factors collectively reduced exposure to raw fish, particularly flounder. In 2023, the number of cases began to rise again, highlighting the need for close monitoring of future trends. Although the measures currently implemented in Japan for farmed and imported flounder appear effective, continued strengthening of surveillance systems and improved reporting consistency are required to accurately monitor trends and prevent future outbreaks.

In this study, approximately half of reported cases occurred among individuals aged 60 years and older, which may be attributed to age-related differences in symptom manifestation, healthcare-seeking behavior, and diagnosis characteristics within the food poisoning surveillance system. Specifically, age-related changes in mucosal immunity, gastric acid secretion, and intestinal barrier function may increase susceptibility to gastrointestinal symptoms among older adults^24^. Thus, infections that remain asymptomatic or mild in younger individuals may be more frequently clinically diagnosed in older adults and consequently captured in surveillance data.

The month with the highest number of cases was October, which supports a previous study reporting several outbreaks in September and October^2^, suggesting seasonal variations. However, the mechanisms underlying this seasonality have not yet been clarified. *K. septempunctata* has been detected year-round, regardless of seawater temperatures^4^. Additionally, because more cases occur in spring in South Korea, the observed seasonal variations are not likely attributable to fluctuations in seawater temperature^4^.

In this study, the average incidence of food poisoning per 1,000,000 population was higher in western Japan and along the Sea of Japan coast. The reason for this is also unclear but may be related to variations in the consumption of sliced raw olive flounder rather than seawater temperature fluctuations^4^. However, no definitive data on fish consumption are currently available.

*Kudoa* species have been documented in fish hosts on all continents except Antarctica^25^. Despite this broad global distribution, studies investigating the role of *Kudoa* spp. in human disease have been conducted primarily in Japan and South Korea, where the consumption of raw fish such as sashimi and sushi is deeply embedded in the food culture. To date, no *Kudoa* spp. infections in humans have been confirmed outside these countries. However, this may reflect underdiagnosis or limited awareness rather than a true lack of disease^26^, and is likely related to the transient nature of symptoms and lack of simple diagnostic methods. Therefore, the development of rapid and convenient diagnostic tools could improve our awareness of this infection in humans. Improving clinician awareness is also important for the accurate recognition, reporting, and prevention of foodborne illness related to *Kudoa* spp.

This study has some limitations. First, under the Food Sanitation Act, physicians are required to report cases only when the illness is considered food poisoning. Owing to incomplete compliance or underreporting^27^, some cases may not have been captured in surveillance data. In addition, when multiple cases occur, local public health centers determine whether an incident should be investigated and classified as a foodborne outbreak. Although such outbreaks are included in national statistics, the threshold for initiating an outbreak investigation varies among local public health centers; therefore, sporadic cases or clusters may not be officially recorded. Furthermore, we focused on food poisoning caused by *K. septempunctata* and did not include cases caused by other *Kudoa* species, such as *Kudoa hexapunctata*^28^. Consequently, the epidemiological characteristics described in this study may underestimate the actual burden of *Kudoa*-related foodborne illness.

Second, the findings of this study may not be generalizable to countries or regions with different food cultures and surveillance systems. In particular, the frequent consumption of raw fish in Japan, such as sashimi and sushi, and the characteristics of the national surveillance and reporting system may limit the applicability of these results to regions where raw seafood consumption is less common or monitoring practices differ.

Third, owing to a lack of prefecture-level data on fish consumption, we did not evaluate the relationship between regional dietary patterns and geographic differences in incidence; thus, we could not distinguish the effects of exposure from those of reporting practices. Finally, detailed clinical information, including disease severity, clinical course, and the origin of implicated fish, was unavailable. Thus, although some food poisoning cases have been linked to live flounder imported from South Korea^1,29^, further assessment was not possible.

In summary, we elucidated the long-term nationwide epidemiological trends of *K. septempunctata* food poisoning in Japan. Most cases were associated with the consumption of flounder sashimi and sushi. The number of reported cases decreased during the COVID-19 pandemic then increased again after the pandemic. Our findings indicate a need to strengthen surveillance systems and improve reporting consistency to better capture the epidemiology of this disease. Future studies are required to examine global epidemiological patterns, global differences in surveillance and reporting systems, and the effectiveness of management and preventive measures. Furthermore, clinicians should consider *Kudoa* infection in the differential diagnosis of food poisoning, particularly when associated with raw fish consumption.

## Supplementary Information

Below is the link to the electronic supplementary material.

Supplementary Material 1

Supplementary Material 2

Supplementary Material 3

## Data Availability

The datasets generated and/or analyzed in this study are available at [https://www.mhlw.go.jp/stf/seisakunitsuite/bunya/kenkou_iryou/shokuhin/syokuchu/04.html].
