# Supplementary material for: Epidemiology of Kudoa septempunctata food poisoning in Japan from 2013 to 2023
Source: Sci Rep. 2026 Feb 9;16:7986. doi: 10.1038/s41598-026-38632-2 (PMC12957298; doi:10.1038/s41598-026-38632-2)
Supplement: Supplementary file 1 — Supplementary Material 1 [file 41598_2026_38632_MOESM1_ESM.docx]

**Epidemiology of *Kudoa septempunctata* Food Poisoning from 2013 to 2023 in Japan**

**SUPPLEMENTARY FILES**

**Supplementary Figure 1**. The number of outbreaks of *Kudoa septempunctata* food poisoning from 2013 to 2023

**Supplementary Figure 2**. Total number of cases of *Kudoa septempunctata* food poisoning reported in Japan by prefecture (2013–2023)
