# Supplementary figures and images for: Epidemiology of Kudoa septempunctata food poisoning in Japan from 2013 to 2023
Source: Sci Rep. 2026 Feb 9;16:7986. doi: 10.1038/s41598-026-38632-2 (PMC12957298; doi:10.1038/s41598-026-38632-2)

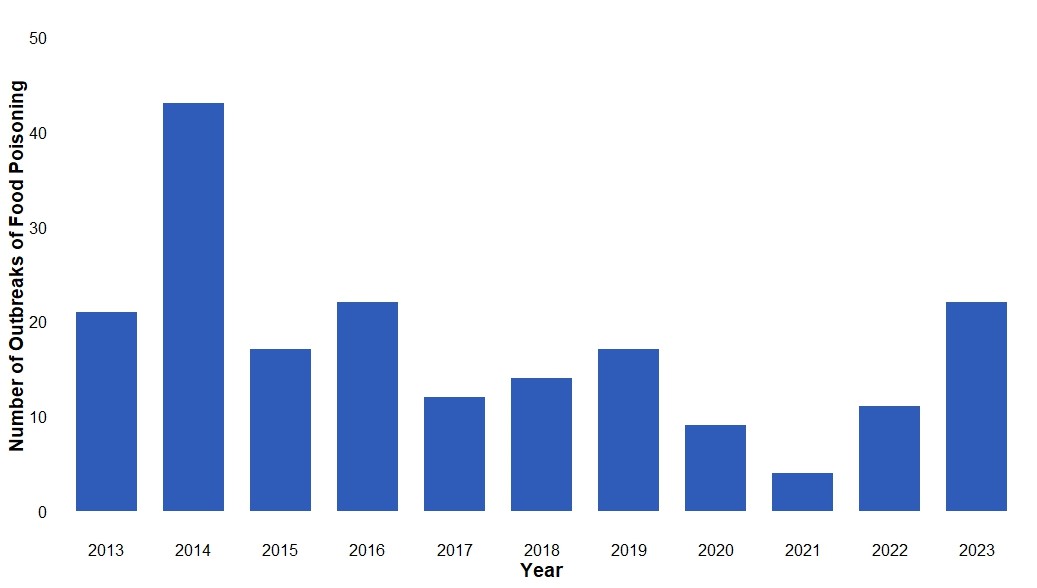

Supplement: Supplementary file 2 — Supplementary Material 2 [file 41598_2026_38632_MOESM2_ESM.jpeg]

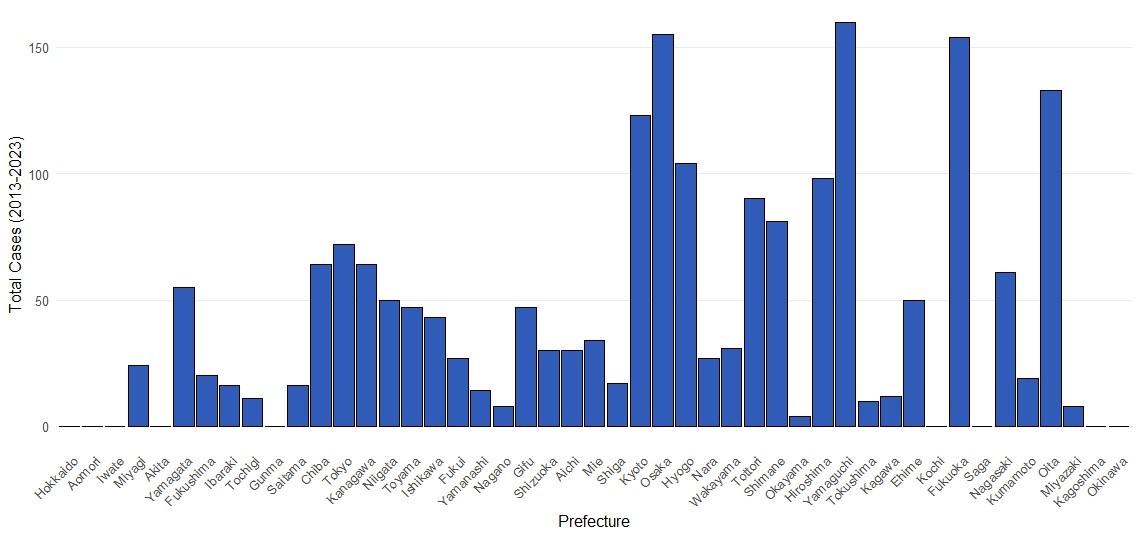

Supplement: Supplementary file 3 — Supplementary Material 3 [file 41598_2026_38632_MOESM3_ESM.jpeg]
